# Determinants of urinary tract infection in hospitalized patients with acute ischemic stroke

**DOI:** 10.1186/s12883-023-03296-2

**Published:** 2023-06-30

**Authors:** Pornpong Jitpratoom, Adhiratha Boonyasiri

**Affiliations:** 1 Division of Medicine, Chumphon Khet Udomsakdi Hospital, Chumphon, Thailand; 2 Department of Research and Development, Faculty of Medicine Siriraj Hospital, Mahidol University, Bangkok, Thailand

**Keywords:** Urinary tract infection, Acute ischemic stroke, Hospitalization, Predictor, Risk factor, Protective factor, Community-acquired, Hospital-acquired, Smoking, Statin

## Abstract

**Background:**

Stroke is a major cause of morbidity and mortality worldwide. Urinary tract infection (UTI) is a common post-acute ischemic stroke (AIS) complication. We assessed the incidence, determinant factors, infection characteristics, post-stroke complications, and outcomes of hospitalized AIS patients with UTI.

**Methods:**

This retrospective cohort study included AIS patients admitted within 7 days of stroke onset. The patients were divided into the UTI group and the non-UTI (control) group. Clinical data were collected and compared between the groups.

**Results:**

There were 342 AIS patients (31 with UTIs and 311 controls). The multivariate analysis showed that an initial National Institutes of Health Stroke Scale (NIHSS) score of ≥ 15 (odds ratio [OR] 5.00, 95% confidence interval [CI] 1.33–18.72) and Foley catheter retention (OR 14.10, 95% CI 3.25–61.28) were risk factors for UTI, whereas smoking (OR 0.08, 95% CI 0.01–0.50), an initial systolic blood pressure (SBP) of > 120 mmHg (OR 0.06, 95% CI 0.01–0.31), and statin use (OR 0.02, 95% CI 0.0006–0.42) were protective factors. Twenty cases (64.5%) were community-acquired and 11 cases (35.3%) were hospital-acquired. Ten patients (32.3%) had catheter-associated UTIs. The most common pathogen was *Escherichia coli* (13 patients, 41.9%). Post-stroke complications were significantly more common in the UTI group, including pneumonia, respiratory failure, sepsis, brain edema, seizure, symptomatic hemorrhagic transformation, congestive heart failure, atrial fibrillation with a rapid ventricular response, acute kidney injury, and hyponatremia. The median length of stay (LOS) in the UTI group was 12 days versus 3 days in the control group (p < 0.001). The median 3-month modified Rankin Scale score was higher (5 in UTI and 2 in control; p < 0.001) and the median 3-month Barthel Index was lower (0 in UTI and 100 in control; p < 0.001) in the UTI group than in the control group.

**Conclusions:**

The risk factors for post-AIS UTI included severe stroke (NIHSS score ≥ 15) and urethral catheter indwelling. An initial SBP of > 120 mmHg and statin use were protective factors. The UTI group had significantly worse post-stroke complications, a longer LOS, and worse 3-month outcomes. Smoking was protective, which requires further investigation.

## Background

Stroke is ranked as the second leading cause of death worldwide. The burden of stroke includes both high mortality and high morbidity, which results in up to 50% of survivors having chronic disability [1, 2]. The injured brain can modify peripheral immunity and transition the functional status of the peripheral immune system from competent to suppressed following the acute phase of stroke [3]. Stroke can cause the transfer and ectopic colonization of specific intestinal flora, making stroke patients more susceptible to infection [4]. Urinary tract infection (UTI) is a common stroke-associated infection [5], with an incidence ranging from 3 to 40% [6–10]. UTIs are associated with poor outcomes [11], death, or disability [7, 12]. Data from previous studies show that the female sex, previous stroke [13], urinary catheterization [14], a higher initial modified Rankin Scale (mRS) score, a higher initial National Institutes of Health Stroke Scale (NIHSS) score [15, 16], older age [12], depressed consciousness, and diabetes mellitus [17] are associated with an increased incidence of infection, but there are many factors that have not been addressed, such as chronic kidney disease, the Barthel Index (BI), the Glasgow Coma Scale (GCS), vital signs, laboratory indicators, and stroke treatments. Therefore, we aimed to assess the determinant factors of UTIs and to explore the incidence, clinical features, and impact of UTIs on stroke outcomes in hospitalized patients with acute ischemic stroke (AIS).

## Methods

This retrospective cohort study was conducted at Chumphon Khet Udomsakdi Hospital in Thailand between July 2019 and July 2020. All hospitalized patients aged ≥ 18 years who presented within 7 days of new-onset AIS were included. Patients were excluded if the information necessary for the study was incomplete. The requirement for informed consent was waived because of the absence of any adverse effect on the rights and welfare of the subjects, as approved by the ethics committee. The diagnosis of UTI was made during hospitalization if the patients developed acute dysuria alone, or fever plus one of the following symptoms: urinary frequency, urgency, or retention; incontinence; gross hematuria; change in urine characteristics; or tenderness in the suprapubic or costovertebral angle, combined with the presence of ≥ 10^3^ of any number of organisms in urine culture [18–22]. All included patients who were not assigned to the UTI group were assigned to the non-UTI (control) group. We classified UTIs into subcategories, with community-acquired UTIs defined as being present within < 48 h of hospital admission and hospital-acquired UTIs defined as being detected following 48 h of hospitalization [23, 24]. Additionally, catheter-associated UTIs (CAUTIs) were defined when patients had an indwelling urinary catheter that had been in place for > 2 days, and either the catheter was in place on the date of infection or removed the day before UTI development [25, 26]. We collected the patients’ demographic data, including age, sex, and vascular risk factors (arterial hypertension, diabetes mellitus, atrial fibrillation, hyperlipidemia, and smoking status). Initial data associated with acute stroke on admission were also collected, including neurological manifestations; laboratory findings, such as complete blood counts, blood urea nitrogen, and creatinine; GCS score, which was used to objectively determine the extent of consciousness impairment and ranged from 3 (coma) to 15 (normal) [27, 28]; NIHSS score, which is a tool for objectively quantifying stroke severity and ranges from 0 (normal) to 42 (coma with quadriplegia) [29, 30]; Trial of Org 10,172 in Acute Stroke Treatment (TOAST) classification [31]; mRS score, which represents the degree of disability because of stroke and ranges from 0 (no symptoms) to 6 (death) [32]; and BI, which was used to measure performance in activities of daily living (ADL), with scores ranging from 0 (totally dependent) to 100 (normal ADL) [33]. Data related to UTI, including urinary tract symptoms, urine analysis (pH, white blood cell count, red blood cell count), urine culture, and Foley catheter status, were also gathered. The impact of UTI on stroke complications, length of stay (LOS), discharge status, 3-month mRS score, and 3-month BI were compiled. To prevent bias, 3-month mRS and BI assessments were blinded to hospital inpatient data, including the history of UTIs. For stroke complications, brain edema was diagnosed when the patient had a new neurological deficit from brain swelling that was seen in a brain image [34]; progressive stroke was defined as gradual worsening of neurological function (NIHSS score increase from admission of ≥ 4) from ongoing ischemic processes [35–37]; and symptomatic intracranial hemorrhage was defined as any hemorrhage with neurologic deterioration, as indicated by an NIHSS score of > 4 points or greater than the baseline value [38].

### Statistical analysis

Descriptive statistics were used to summarize the demographic features, such as patient age and sex. Quantitative data are presented as the mean ± standard deviation or median (interquartile range [IQR]), and qualitative data are presented as frequency (percentage). The chi-square test or Fisher’s exact test was performed on categorical variables, such as patient sex, across the control and UTI groups. LOS, complications, and treatment outcomes were compared between the two groups using Fisher’s exact test and the Mann–Whitney U test. Multinomial logistic regression was used to estimate the odds ratios (ORs) and 95% confidence intervals (CIs) of the control and UTI groups via a manual backward step. Factors with p values of < 0.1 in the univariate analysis were considered for inclusion in the multivariate analysis. Multiple logistic regression based on the backward elimination method with removal of variables with p values of > 0.2 was used for the adjusted model. All statistical analyses were performed using the PASW Statistics 18.0 package (Predictive Analytics Software, SPSS Inc., Chicago, IL, US).

### Ethics approval

This study was approved by the human research ethics committee of the Faculty of Medicine, Thammasat University (Ref: MTU-EC-OO-0-124/63). All methods were performed in accordance with relevant guidelines and regulations, such as the Declaration of Helsinki, the Belmont Report, Council for International Organizations of Medical Sciences guidelines, and ICH-Good Clinical Practice guidelines.

## Results

### Demographic features of patients with AIS

**Fig. 1:**
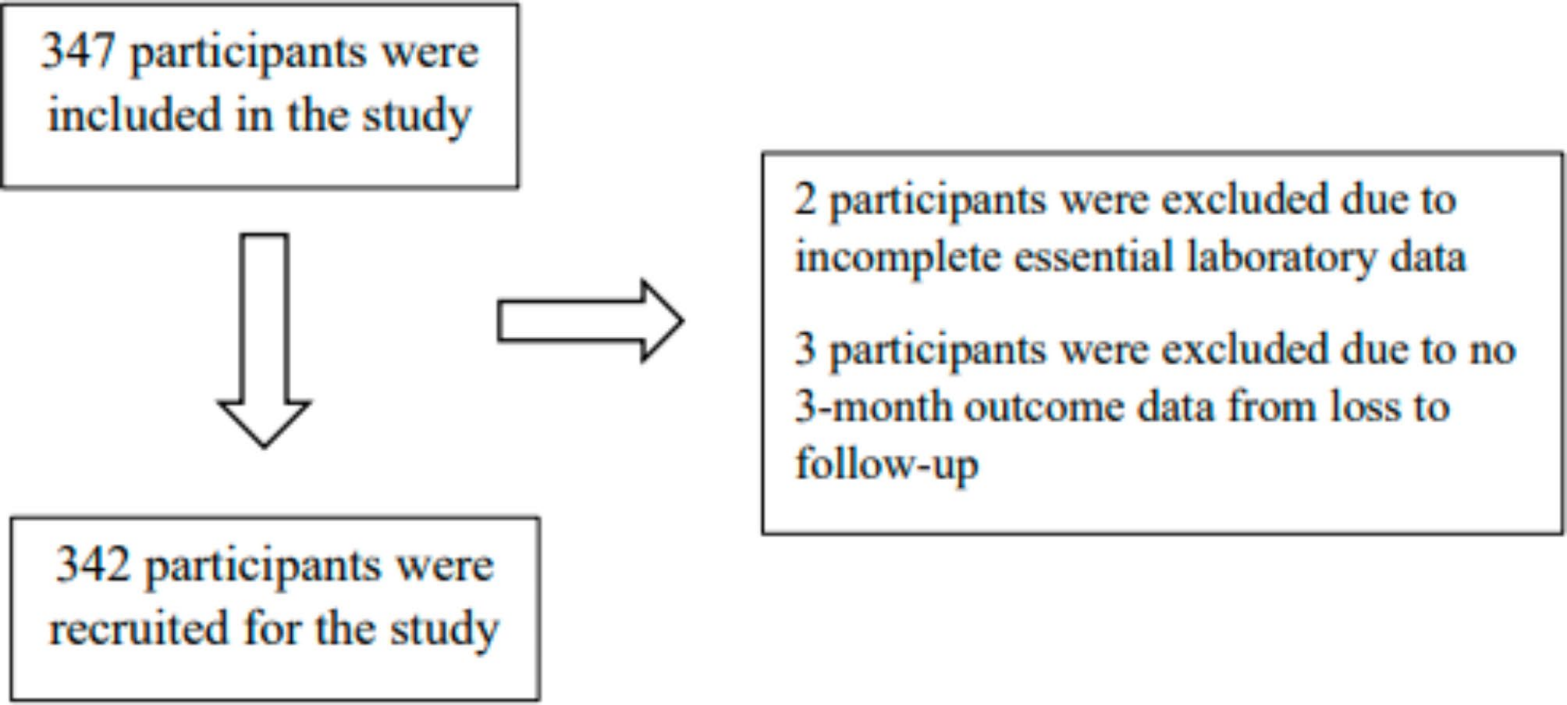
Flow diagram showing study recruitment

A total of 342 hospitalized patients with AIS were recruited (Fig. 1). The median age (IQR) of the patients was 66 years, and 61% of the patients were male. The most common atherosclerotic risk factor was hypertension (72%), followed by dyslipidemia (54%), smoking (41%), obesity according to a body mass index of ≥ 25 kg/m^2^ (36%), and diabetes mellitus (30%). The patients were categorized according to the TOAST classification into five subtypes: (1) large-artery atherosclerosis (30%); (2) cardioembolism (19%); (3) small-vessel occlusion (46%); (4) stroke of other determined etiology (3%); and (5) stroke of undetermined etiology (1%). The lesion was in the anterior circulation in 248 patients (73%), in the posterior circulation in 77 patients (23%), and unknown (diagnosis of AIS without a definite lesion on imaging) in 17 patients (5%).

### Factors associated with UTI

Patients were classified into two groups. If they were diagnosed with UTI during hospitalization, they were assigned to the UTI group (31 patients, 9%). Otherwise, they were assigned to the control group (311 patients, 91%). The univariate analysis of factors associated with UTI is shown in Table 1. The final multivariate analysis showed that an initial NIHSS score of ≥ 15 (OR 5.00, 95% CI 1.33–18.72) and Foley catheter retention (OR 14.10, 95% CI 3.25–61.28) were risk factors for infection, whereas smoking (OR 0.08, 95% CI 0.01–0.50), an initial systolic blood pressure (SBP) of > 120 mmHg (OR 0.06, 95% CI 0.01–0.31), and statin use (OR 0.02, 95% CI 0.0006–0.42) were protective factors (Table 2).

**Table 1:** Univariate analysis of factors influencing UTI

Variables	Total (n = 342)	Controls (n = 311)	UTI (n = 31)	p value
**Sex**				
Male, n (%)	207 (60.5)	191 (61.4)	16 (51.6)	0.287
Age (years), median (IQR)	66 (54, 77)	65 (54, 76)	77 (63, 84)	0.002
Age ≥ 70, n (%)	141 (41.2)	121 (38.9)	20 (64.5)	0.006
**Risk factors for stroke**				
Alcoholism, n (%)	87 (25.4)	83 (26.7)	4 (12.9)	0.093
Smoking, n (%)	140 (40.9)	134 (43.1)	6 (19.4)	0.010
Hypertension, n (%)	246 (71.9)	225 (72.3)	21 (67.7)	0.59
Diabetes mellitus, n (%)	101 (29.5)	94 (30.2)	7 (22.6)	0.37
Chronic kidney disease, n (%)	79 (23.1)	68 (21.9)	11 (35.5)	0.086
Dyslipidemia, n (%)	185 (54.1)	167 (53.7)	18 (58.1)	0.64
Coronary artery disease, n (%)	42 (12.3)	34 (10.9)	8 (25.8)	0.038
Atrial fibrillation, n (%)	62 (18.1)	47 (15.1)	15 (48.4)	< 0.001
Previous stroke, n (%)	52 (15.2)	47 (15.1)	5 (16.1)	0.80
**Neurological manifestations**				
Alteration of consciousness, n (%)	59 (17.3)	42 (13.5)	17 (54.8)	< 0.001
Weakness, n (%)	303 (88.6)	272 (87.5)	31 (100)	0.035
Vertigo, n (%)	58 (17.0)	57 (18.3)	1 (3.2)	0.033
Aphasia, n (%)	54 (15.8)	44 (14.1)	10 (32.3)	0.017
**Initial vital signs on admission**				
SBP, mean ± SD (mmHg)	160 ± 31	162 ± 31	146 ± 31	0.006
SBP > 120 mmHg, n (%)	306 (89.5)	284 (91)	22 (71)	0.002
DBP, mean ± SD (mmHg)	92 ± 20	93 ± 20	83 ± 19	0.013
h, mean ± SD (beats/minute)	80 ± 19	80 ± 18	87 ± 26	0.043
Temperature, mean ± SD (°C)	36.8 ± 0.4	36.8 ± 0.4	37.0 ± 0.5	0.007
RR, mean ± SD (breaths/minute)	21 ± 3	21 ± 3	22 ± 5	0.034
**Laboratory indicators**				
Glucose, mean ± SD (mg/dL)	145 ± 76	144 ± 71	157 ± 117	0.69
BUN, mean ± SD (mg/dL)	16 ± 10	16 ± 9	21 ± 13	0.005
Creatinine, mean ± SD (mg/dL)	1 ± 0.6	1 ± 0.7	1 ± 0.4	0.45
Hemoglobin, mean ± SD (g/dL)	13 ± 2	13 ± 2	12 ± 2	0.092
White blood cell count, mean ± SD (cells/mm^3^)	9,138 ± 3,384	9,047 ± 3,255	10,058 ± 4,443	0.11
Neutrophil count, mean ± SD (%)	64 ± 14	63 ± 14	70 ± 13	0.006
Lymphocyte count, mean ± SD (%)	25 ± 12	26 ± 12	20 ± 10	0.005
**Initial score on admission**				
mRS score, median (IQR)	4 (4, 5)	4 (3, 5)	5 (5, 5)	< 0.001
BI, median (IQR)	50 (25, 70)	55 (35, 70)	10 (0, 35)	< 0.001
GCS score, median (IQR)	15 (13, 15)	15 (14, 15)	10 (7, 13)	< 0.001
NIHSS score, median (IQR)	6 (3, 14)	5 (3, 12)	22 (15, 28)	< 0.001
NIHSS score ≥ 15, n (%)	83 (24.3)	58 (18.6)	25 (80.6)	< 0.001
**Treatment**				
Thrombolysis (rt-PA), n (%)	47 (13.7)	40 (12.9)	7 (22.6)	0.17
Antiplatelets, n (%)	281 (82.2)	264 (84.9)	17 (54.8)	< 0.001
Anticoagulants, n (%)	52 (15.2)	40 (12.9)	12 (38.7)	< 0.001
Statins, n (%)	336 (98.2)	308 (99.0)	28 (90.3)	0.011
**Devices**				
Retained Foley catheter, n (%)	88 (25.7)	62 (19.9)	26 (80.6)	< 0.001
NG tube placement, n (%)	82 (24.0)	56 (18.0)	26 (80.6)	< 0.001
Failed WST, n (%)	85 (24.9)	59 (19.0)	26 (83.9)	< 0.001

BI, Barthel Index; BPH, benign prostatic hyperplasia; BUN, blood urea nitrogen; cells/mm^3^, cells per milliliter; DBP, diastolic blood pressure; GCS, Glasgow Coma Scale; g/dL, grams per deciliter; GFR, glomerular filtration rate; HR, heart rate; ml/min, milliliters per minute; IQR, interquartile range; mg/dL, milligrams per deciliter; mmHg, millimeters of mercury; mRS, modified Rankin Scale; n, number; NG, nasogastric; NIHSS, National Institutes of Health Stroke Scale; p value, comparisons between the control group and the urinary tract infection group; RR, respiratory rate; rt-PA, recombinant tissue plasminogen activator; SBP, systolic blood pressure; SD, standard deviation; UTI, urinary tract infection; WST, water-swallowing test

**Table 2:** Multivariate logistic regression analysis of factors influencing UTI

Variables	OR (95% CI)	p value
Age ≥ 70 years	3.51 (0.77–16.06)	0.106
Smoking	0.08 (0.01–0.50)	0.007
**Underlying diseases**		
Chronic kidney disease	0.35 (0.08–1.47)	0.150
**Initial vital sign**		
Systolic blood pressure > 120 mmHg	0.06 (0.01–0.31)	0.001
**Initial scoring system**		
Initial GCS score ≤ 8	4.18 (0.79–22.18)	0.093
Initial NIHSS score ≥ 15	5.00 (1.33–18.72)	0.017
**Treatment**		
Antiplatelets	4.82 (0.45–51.44)	0.193
Anticoagulants	7.86 (0.69–89.86)	0.097
Statins	0.02 (0.0006–0.42)	0.013
**Device**		
Retained Foley catheter	14.10 (3.25–61.28)	< 0.001

OR, odds ratio; CI, confidence interval; GCS, Glasgow Coma Scale; mmHg, millimeters of mercury; NIHSS, National Institutes of Health Stroke Scale; p value, comparisons between the control group and the urinary tract infection group

### Characteristics of UTIs

Of the 31 patients with UTIs, 20 cases (64.5%) were community-acquired and 11 cases (35.3%) were hospital-acquired. Ten patients (32.3%) had CAUTIs. The three most common UTI symptoms included fever (96.8%), turbid urine (45.2%), and urinary retention (41.9%). In 1991, the Sepsis-1 diagnostic criteria were established, and included infection or suspected infection, leading to onset of systemic inflammatory response syndrome (SIRS). SIRS criteria to identify sepsis have also been established, and include primarily tachycardia (heart rate of > 90 beats/min); tachypnea (respiratory rate of > 20 breaths/min); fever or hypothermia (temperature of > 38 °C or < 36 °C); and leukocytosis, leukopenia, or bandemia (white blood cell count of > 1,200/mm^3^ or < 4,000/mm^3^; bandemia of ≥ 10%). If patients met two or more of these criteria, they fulfilled the definition of SIRS [39]. In the present study, the median (IQR) of the SIRS criteria in patients with UTI was 3. Subsequently, a 2016 proposal defined the Sepsis-3 criteria as life-threatening organ dysfunction caused by a dysregulated host response to infection [40–42]. The quick Sepsis-Related Organ Failure Assessment (qSOFA) was designed to rapidly diagnose suspected sepsis, and it consists of three components, each worth 1 point, including a respiratory rate of ≥ 22 breaths/min; change in mental status; and SBP of ≤ 100 mmHg. A qSOFA score of 2 indicates organ dysfunction [43]. The median (IQR) qSOFA score in the present study was 1. Secondary bacteremia was observed in only two patients (6.5%). The three most common pathogens were *Escherichia coli (E. coli)* (41.9%), *Klebsiella pneumoniae* (16.1%), and *Enterococcus faecalis* (12.9%). The antibiotic susceptibility pattern of UTI-causative organisms was assessed. The susceptibility pattern of *E. coli* showed good sensitivity (> 80% susceptibility) to amoxicillin/clavulanic acid, third-generation cephalosporins, piperacillin/tazobactam, and carbapenems. Over 30% of *E. coli* were resistant to gentamicin, trimethoprim/sulfamethoxazole, and quinolones. The antibiotic susceptibility of *E. coli* and the data described above are shown in Table 3. Most patients underwent antibiotic monotherapy, while only seven (23.3%) underwent combination therapy.

**Table 3:** Characteristics of UTIs

Characteristics of UTIs	Total (n = 31)
**Type of infection**	
• Community-acquired, n (%)	20 (64.5)
• Hospital-acquired, n (%)	11 (35.5)
Catheter-associated urinary tract infection, n (%)	10 (32.3)
Duration of retained Foley catheter since admission (days), median (IQR)	12 (5, 28.75)
Time to acquire urinary tract infection (days), median (IQR)	2 (1, 7)
**Symptoms**	
• Fever, n (%)	30 (96.8)
• Turbid urine, n (%)	14 (45.2)
• Urinary retention, n (%)	13 (41.9)
• Dysuria, n (%)	6 (19.4)
• Urinary incontinence, n (%)	2 (6.5)
• Gross hematuria, n (%)	1 (3.2)
SIRS criteria (points), median (IQR)	3 (2, 4)
qSOFA (points), median (IQR)	1 (1, 2)
Secondary bacteremia, n (%)	2 (6.5)
**Pathogens**	
• *Escherichia coli*, n (%)	13 (41.9)
• *Klebsiella pneumoniae*, n (%)	5 (16.1)
• *Enterococcus faecalis*, n (%)	4 (12.9)
• *Acinetobacter baumannii*, n (%)	3 (9.7)
**Antibiotics susceptible to** ***Escherichia coli*** **(N = 13)**	
• Amoxicillin/clavulanic acid, n (%)	11 (84.6)
• Ceftriaxone, n (%)	11 (84.6)
• Ceftazidime, n (%)	11 (84.6)
• Cefepime, n (%)	11 (84.6)
• Gentamicin, n (%)	8 (61.5)
• Amikacin, n (%)	13 (100)
• Ciprofloxacin, n (%)	5 (38.5)
• Trimethoprim/sulfamethoxazole, n (%)	9 (69.6)
• Piperacillin/tazobactam, n (%)	12 (92.3)
• Ertapenem, n (%)	13 (100)
• Meropenem, n (%)	13 (100)
**Treatment (N = 30)**	
Monotherapy, n (%)	23 (76.7)
• Ceftriaxone, n (%)	11 (36.7)
• Ceftazidime, n (%)	5 (16.7)
• Piperacillin/tazobactam, n (%)	7 (23.3)
Combination therapy, n (%)	7 (23.3)

IQR, interquartile range; n, number; N, number of patients who underwent antimicrobial susceptibility test; qSOFA, quick Sepsis-Related Organ Failure Assessment; SIRS, systemic inflammatory response syndrome; UTI, urinary tract infection

### Complications following stroke and treatment outcomes

As shown in Table 4, the proportion of various post-stroke complications was significantly higher in the UTI group than in the control group, such as pneumonia, respiratory failure, sepsis, brain edema, seizure, symptomatic hemorrhagic transformation, congestive heart failure, atrial fibrillation with a rapid ventricular response, acute kidney injury, and hyponatremia. The median LOS of patients in the UTI group was higher than in the control group (12 days vs. 3 days, respectively; p < 0.001). In terms of treatment outcomes, the proportion of patients without an improvement in discharge status and who died was higher in the UTI group (29% and 29%, respectively) than in the control group (5% and 3%, respectively). Regarding 3-month stroke outcomes, the mRS and BI scores, for which extensive evidence exists supporting their use as assessments of stroke outcomes, were poorer in the UTI group [32, 33]. Significantly higher median 3-month mRS scores (5 vs. 2, p < 0.001) and lower median 3-month BI scores (0 vs. 100, p < 0.001) were observed in the UTI group. With a significant p value of < 0.001, 87.1% of patients in the UTI group had a 3-month mRS score of ≥ 3, which indicates an unfavorable outcome in the assessment of disability [44–46], compared with 43.7% in the control group. The UTI group had a higher percentage of patients with a 3-month BI score of ≤ 40 than the control group (83.9% vs. 22.2%, respectively; p < 0.001). A BI score of ≤ 40 implies that performance in ADL is completely dependent on others [33].

**Table 4:** Complications, length of stay, discharge status, and 3-month outcomes of patients with and without UTIs

Variables	Total (n = 342)	Controls (n = 311)	UTI (n = 31)	p value
**Complications**				
Pneumonia, n (%)	62 (18)	40 (13)	22 (71)	< 0.001
Respiratory failure, n (%)	42 (12)	23 (7)	19 (61)	< 0.001
Sepsis, n (%)	64 (19)	36 (12)	28 (90)	< 0.001
Brain edema, n (%)	12 (4)	5 (2)	7 (23)	< 0.001
Seizure, n (%)	14 (4)	10 (3)	4 (13)	0.029
Progressive stroke, n (%)	10 (3)	10 (3)	0 (0)	0.61
Symptomatic hemorrhagic transformation, n (%)	7 (2)	2 (1)	5 (16)	< 0.001
Asymptomatic hemorrhagic transformation, n (%)	8 (2)	6 (2)	2 (6)	0.16
Gastrointestinal bleeding, n (%)	11 (3)	9 (3)	2 (6)	0.26
Myocardial infarction, n (%)	3 (1)	2 (1)	1 (3)	0.25
Congestive heart failure, n (%)	21 (6)	16 (5)	5 (16)	0.031
Atrial fibrillation with rapid ventricular response, n (%)	11 (3)	6 (2)	5 (16)	0.001
Hypoglycemia, n (%)	5 (1)	4 (1)	1 (3)	0.38
Hyperglycemia, n (%)	3 (1)	2 (1)	1 (3)	0.25
Acute kidney injury, n (%)	21 (6)	13 (4)	8 (26)	< 0.001
Hyponatremia, n (%)	9 (3)	5 (2)	4 (13)	0.005
**Length of stay, median (IQR)**	3 (2, 5)	3 (2, 4)	12 (6, 29)	< 0.001
**Discharge status**				< 0.001
Complete recovery, n (%)	10 (3)	10 (3)	0 (0)	
Improvement, n (%)	288 (84)	275 (88)	13 (42)	
No improvement, n (%)	25 (7)	16 (5)	9 (29)	
Death, n (%)	19 (6)	10 (3)	9 (29)	
**3-month outcome**				
mRS score, median (IQR)	2 (1, 4)	2 (1, 4)	5 (5, 6)	< 0.001
mRS score ≥ 3, n (%)	163 (47.7)	136 (43.7)	27 (87.1)	< 0.001
BI score, median (IQR)	95 (35, 100)	100 (55, 100)	0 (0, 10)	< 0.001
BI score ≤ 40, n (%)	95 (27.8)	69 (22.2)	26 (83.9)	< 0.001

BI, Barthel index; IQR, interquartile range; mRS, modified Rankin Scale; n, number; p value, comparisons between the control group and the urinary tract infection group; UTI, urinary tract infection

## Discussion

In this retrospective cohort trial of the determinants of UTI in hospitalized patients with AIS, we discovered that an initial NIHSS score of ≥ 15 on admission and Foley catheter retention during hospitalization were aggravating factors for infection. However, previous smoking, an initial SBP of > 120 mmHg on admission, and statin use were preventive factors.

The incidence of UTI in our study was 9% (31 patients), which is congruent with previous studies reporting incidence rates of 3.7–19% [13–15, 17, 47, 48]. According to the multivariate analysis in the present study, an NIHSS score of ≥ 15 on first admission is associated with an increased risk of UTI. Consistent with data reported by Li et al. in 2020, a higher NIHSS score was associated with UTI. Additionally, in 2022, Mukapa et al. found that an NIHSS score of 16–42 is a risk for UTI (OR 5.15, 95% CI 1.68–15.75; p < 0.001) [15, 16]. Therefore, stroke severity, as evaluated using the NIHSS on admission, appears to be associated with the incidence of UTI.

Foley catheter retention is a well-established risk factor for healthcare-associated UTI. Moreover, AIS is associated with a high incidence of bladder dysfunction. These conditions have been observed in patients with large infarcts and in those with infarcts located in the cortex, which increase the likelihood of catheterization, thereby further increasing the risk of UTI [49, 50]. CAUTIs are a leading cause of secondary nosocomial bloodstream infection [51]. According to a 2009 report by Stott et al., post-stroke UTI is associated with urinary catheterization (OR 3.03, 95% CI 1.41–6.52) [12], which is comparable to the observation of the present study.

We identified smoking as a protective factor. Cigarette smoking is a well-known major risk factor for premature mortality because of cancer, cardiovascular disease, and chronic obstructive pulmonary disease. Smoking appears to be a risk factor for respiratory tract infections, although the risk of UTIs with smoking is unknown [52, 53]. According to a 2001 study by Alnaif et al. (in women) and a 2018 study by Yongzhi et al. (in patients with urolithiasis), smoking is not associated with UTIs [54, 55]. However, smoking may contribute to the development of lower urinary tract symptoms in women [56]. Despite these results, it should be noted that no related studies have previously been conducted in the post-stroke population. The effects of smoking on urinary tract microorganisms have been studied, and it was found that microorganism species differed between smokers and non-smokers [57]. The urobiota plays a role in bladder homeostasis by maintaining the integrity of the urinary tract epithelium, protecting against infection, regulating neurotransmission, and promoting proper immune system functioning, which affects the occurrence of UTIs [58, 59]. Accordingly, smoking may influence UTIs through this mechanism.

Another protective factor was an initial SBP of > 120 mmHg on admission, which can be explained by the fact that known UTIs can cause urosepsis, and approximately one third of these patients present with hypotension [60]. Moreover, when stroke occurs, blood pressure often increases because of various factors, including psychological stress, pain, urinary retention, and hypoxemia [61]. In addition, stroke can lead to permanent damage to the areas of the brain involved in the regulation of cardiovascular functioning, including blood pressure. An acute hypertensive response is observed in > 60% of patients with AIS according to previous studies [62–64]. Therefore, the relative hypotension on admission from occult urosepsis, which is particularly common with community-acquired UTIs (64.5%) that present within 48 h, can be just within the normal range of SBP (the mean initial SBP of the control and UTI groups was 162 mmHg and 146 mmHg, respectively). In contrast, high blood pressure may be a protective factor under these conditions. A review of previous literature revealed that initial blood pressure has not previously been examined in the context of the associations between UTIs and acute stroke [12–17, 48, 50, 65–67]. Therefore, this is the first report of this finding.

The present study supports the observation that statin use is a preventive factor for UTIs. Preclinical studies have indicated that statins can indeed reduce bacterial invasion [68–71]. In 2013, Pouwels et al. found that pravastatin can reduce the occurrence of recurrent UTIs [72]. The postulated mechanism is that bacterial invasion of the bladder epithelium requires the membrane-bound protein Rac1, the expression of which can be reduced by statins [71, 73, 74].

In our study, 64.5% of UTIs were community-acquired, 35.3% were hospital-acquired, and 32.3% were CAUTIs, which approximates the findings of Bogason et al. from 2017 [14]. The causative organisms most commonly involved in UTIs are those found in the colonic bacterial flora, primarily *E. coli*, which may be because of their capacity to attach to the uroepithelium. However, other Enterobacterales are also common [16, 75]. The high prevalence of *E. coli* was comparable to that reported in previous studies [16, 65, 67].

In 2019, the National Antimicrobial Resistant Surveillance in Thailand reported that *E. coli* urinary isolates from outpatients were susceptible to ciprofloxacin (30.1%), cotrimoxazole (42.2%), ceftriaxone (59.7%), and amikacin (98.8%) [76]. The susceptibility patterns to ciprofloxacin, cotrimoxazole, and amikacin were similar to those in our study, whereas the susceptibility to ceftriaxone (59.7%) was lower than in our study (84.6%). When we compared the antibiotic susceptibility patterns of *E. coli* in the present study with the antibiotic susceptibility patterns of community-acquired infections from another tertiary care hospital in the same region of Thailand within the same period [77], both antibiotic susceptibility patterns were similar, and low-level resistance to third-generation cephalosporins, amikacin, and carbapenems was observed. Therefore, according to the present study, third-generation cephalosporins remain an effective treatment option for post-stroke UTIs in Thailand. However, in other countries, the susceptibility of *E. coli* to third-generation cephalosporins in the context of UTI varies between 53.5% and 94.9%. As a result, antibiotic treatment should be based on the local antibiogram [78–83].

Various post-stroke complications were more prevalent in the UTI group than in the control group in the present study. These included infection; respiratory failure; bleeding; and neurological, cardiovascular, metabolic, and renal complications. According to our review of the literature, no studies have addressed stroke complications in such patients [12–17, 48, 50, 65–67, 84, 85]. Sepsis can be caused by UTIs [86], and the severity of the UTIs appears to be increasing and is typically associated with hyponatremia [87, 88], acute respiratory distress syndrome [86] leading to respiratory failure [89], disseminated intravascular coagulopathy [86] leading to bleeding complications [90], sepsis-induced cardiomyopathy leading to heart failure [91], atrial fibrillation [92] leading to atrial fibrillation with a rapid ventricular response [93, 94], seizure [95], and acute kidney injury [86]. UTIs increase the odds of a decline in neurological status during hospitalization [11], and severe neurologic deficit increases the risk of stroke-associated pneumonia [96]. Although rare, UTIs can spread to the lungs, causing pneumonia [97]. One limitation of our study is that we did not document the temporal relationship between brain edema and UTIs. Brain edema is a severe stroke complication that is associated with prolonged hospitalization and poor outcomes [98, 99]. AIS with cerebral edema usually requires urinary catheterization for intensive urine output monitoring [100] and prolonged immobility, which can increase the risk of UTI [101]. This may be the reason why the UTI group had a greater number of patients with cerebral edema than the control group. Several studies have shown a longer LOS in the UTI population than in the control population [13, 14, 16, 50, 65]. Patients with UTI also have a poorer discharge status [16, 50] and 3-month outcomes [7, 12, 66], which is consistent with our study.

Although stroke severity itself may increase the risk of UTI [15, 16] and could explain some of the associations between UTIs and poorer outcomes, a causal effect of UTI on stroke recovery is also possible and may be multifactorial. It is well documented that fever in patients with brain injury of any etiology, including stroke, correlates with poorer outcomes in many measures, including mortality, mRS score, BI, and hospital LOS [102]. Additionally, increasing evidence from animal studies suggests that systemic inflammatory insults that simulate infection promote antigen presentation and autoimmunity against the brain, which are key elements in the pathobiology of stroke, and may also play a central role related to the clinical impact of post-stroke infection [103, 104]. This may lead to prolonged damaging cytokine production and microgliosis, which can have a profound effect on the onset and/or progression of neurodegenerative diseases [105–108], and these responses are associated with poorer stroke outcomes. However, evidence pertaining to this issue is scarce, and the exact pathophysiology remains to be investigated.

This study has some limitations that should be noted when interpreting the results. Only patients from hospitals providing tertiary care participated in the study. Therefore, theoretically, our findings may not be applicable to other populations receiving care at different facilities. Moreover, the number of patients included in this study was relatively small, resulting in unavoidable selection bias.

## Conclusions

UTIs are a major infectious complication in hospitalized patients with AIS. Stroke severity and Foley catheter retention were re-established as risk factors for post-stroke UTI, while an initial SBP of > 120 mmHg and statin use were newly recognized as protective factors that have not previously been described in this population. Smoking was associated with an increased risk of infection in many organs, particularly the respiratory tract. However, we found smoking to be another protective factor against UTI, which has not previously been reported in such patients. Accordingly, this phenomenon requires further study. Two thirds of all UTIs were community-acquired, and *E. coli* was the predominant pathogen. For the first time, we showed that many post-stroke complications (e.g., pneumonia, seizure, congestive heart failure, and acute kidney injury) in patients with AIS with UTI are significantly more frequent than in patients without UTI. Finally, the longer LOS and poorer outcomes of patients with UTI were reconfirmed in this study.

## Data Availability

Individual level data cannot be shared publicly because of patient confidentiality under current Thai legislation. The data that support the findings of this study are available from Chumphon Khet Udomsakdi Hospital, but restrictions apply to the availability of these data, which were used under license for the current study, and so are not publicly available. Data are however available from Dr. Pornpong Jitpratoom (Email: pornpong31@gmail.com) upon reasonable request and with permission of Chumphon Khet Udomsakdi Hospital and the appropriate ethics committee.

## Abbreviations

ADL: activities of daily living
AIS: Acute Ischemic Stroke
BI: Barthel Index
BPH: Benign Prostatic Hyperplasia
BUN: Blood Urea Nitrogen
cells/mm^3^: cells per milliliter
CI: Confidence Interval
CIOMS: Council for International Organizations of Medical Sciences
DBP: Diastolic Blood Pressure
*E. coli*: *Escherichia coli*
GCS: Glasgow Coma Scale
g/dL: grams per deciliter
GFR: Glomerular Filtration Rate
HR: Heart Rate
ICH-GCP: International Conference on Harmonization-Good Clinical Practice
IQR: interquartile range
LOS: length of stay
ml/min: milliliters per minute
mg/dL: milligrams per deciliter
mmHg: millimeters of mercury
mRS: modified Rankin Scale
n: number
NG tube: Nasogastric tube
NIHSS: National Institutes of Health Stroke Scale
OR: Odds Ratio
qSOFA: quick Sepsis-related Organ Failure Assessment
RR: respiratory rate
rt-PA: recombinant tissue Plasminogen Activator
SBP: Systolic blood pressure
SD: standard deviation
SIRS: systemic inflammatory response syndrome
SPSS: Statistical Package for the Social Sciences
TOAST: Trial of ORG 10172 in Acute Stroke Treatment
UTI: Urinary Tract Infection
USA: United States of America
WST: Water-Swallowing Test
